# Population genetic structure and morphological diversity of *Cruzia tentaculata* (Nematoda: Ascaridida), a parasite of marsupials (Didelphinae), along the Atlantic Forest on the eastern coast of South America

**DOI:** 10.1017/S0031182022000981

**Published:** 2022-09

**Authors:** Renata Souza, Roberto do Val Vilela, Rosana Gentile, Eduardo José Lopes-Torres, Pedro Cordeiro-Estrela, Ricardo Moratelli, Sócrates Fraga da Costa-Neto, Thiago dos Santos Cardoso, Karina Varella, Arnaldo Maldonado Júnior

**Affiliations:** 1 Programa de Pós-Graduação em Biodiversidade e Saúde, Instituto Oswaldo Cruz, Fundação Oswaldo Cruz, Av. Brasil, 4365, 21040-360, Rio de Janeiro, RJ, Brasil; 2 Laboratório de Biologia e Parasitologia de Mamíferos Silvestres Reservatórios, Instituto Oswaldo Cruz, Fundação Oswaldo Cruz, Av. Brasil 4365, 21040-360, Rio de Janeiro, RJ, Brasil; 3 Laboratório de Helmintologia Romero Lascasas Porto, Departamento de Microbiologia, Imunologia e Parasitologia, Faculdade de Ciências Médicas, Centro Biomédico, Universidade do Estado do Rio de Janeiro – UERJ, Rio de Janeiro, RJ, Brasil; 4 Departamento de Sistemática e Ecologia, Laboratório de Mamíferos, Programa de Pós-graduação em Ciências Biológicas, Universidade Federal da Paraíba, Jardim Universitário, s/n, Castelo Branco III, CEP 58051-900, João Pessoa, PB, Brasil; 5 Fiocruz Mata Atlântica, Fundação Oswaldo Cruz, Av. Sampaio Corrêa s/n, 22713-560, Rio de Janeiro, RJ, Brasil; 6 Programa de Pós-Doutorado Nota 10–2021, FAPERJ – Fundação Carlos Chagas Filho de Amparo à Pesquisa do Estado do Rio de Janeiro, Av. Erasmo Braga, 118, 20020-000, Rio de Janeiro, RJ, Brasil; 7 Programa de Pós-Graduação em Biologia Parasitária (PPGBP), Instituto Oswaldo Cruz, Fundação Oswaldo Cruz, Manguinhos, Rio de Janeiro, RJ, Brazil

**Keywords:** AMOVA, fixation index, Kathlaniidae, Mantel test, morphology, nematode, neutrality tests, opossum, population genetics

## Abstract

*Cruzia tentaculata* is a helminth parasite of marsupials and has a wide geographic distribution from Mexico to Argentina. The aim of this study was to analyse the genetic population structure of this nematode along the Atlantic Forest biome. *Cruzia tentaculata* specimens were recovered from *Didelphis aurita*, *Didelphis albiventris* and *Philander quica* in 9 localities. Morphological and morphometric data were investigated for phenotypic diversity among localities and hosts using multivariate discriminant analysis of principal components. Phylogenetic relationships of *C. tentaculata* were determined using maximum likelihood and Bayesian inference. The population structure was analysed by fixation indices, molecular variance analysis, Tajima's *D* and Fu's *F*s neutrality tests, Mantel tests and Bayesian clustering analysis. A higher significant morphometric difference for males was observed between localities. In the haplogroup networks, 2 groups were recovered, separating locations from the north and from the south/southeast. The morphometric variation in *C*. *tentaculata* between different localities was compatible with this north and southeast/south pattern, suggesting adaptation to different ecological conditions. Population genetic analyses suggested a pattern of evolutionary processes driven by Pleistocene glacial refugia in the northeast and southeast of the Atlantic Forest based on the distribution of genetic diversity.

## Graphical abstract

**Figure:**
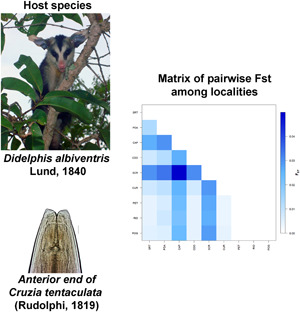

## Introduction

The genus *Cruzia* Travassos, 1917 comprises 10 species that parasitize the caecum and large intestine mainly of mammals but also amphibians and reptiles (Sprehn, 1932; Ruiz, 1947; Wolfgang, 1951; Crites, 1956; Wahid, 1964; Costa, 1965; Ubelaker and Younus, 1965; Guerrero, 1971; Adnet *et al*., 2009; Vieira *et al*., 2020). Up to now, 3 species of *Cruzia* have been reported infecting marsupials in the Americas: *Cruzia cameroni* Wolfgang, 1951 infecting *Didelphis marsupialis* Linnaeus, 1758 in Trinidad, *Cruzia americana* Maplestone, 1930 infecting *D. marsupialis* and *Didelphis virginiana* in the USA and *Cruzia tentaculata* Travassos, 1917 infecting *Caluromys philander* (Linnaeus, 1758), *Didelphis albiventris* Lund, 1841, *Didelphis aurita* Wied-Neuwied, 1826, *D. marsupialis*, *Marmosa murina* (Linnaeus, 1758), *Metachirus myosuros* (Temminck, 1824), *Metachirus nudicaudatus* (Geoffroy, 1803), *Monodelphis domestica*, Wagner, 1842 and *Philander opossum* Linnaeus, 1758, with the larger geographic distribution, occurring in Argentina, Bolivia, Brazil, Colombia, Paraguay, Peru, French Guiana and Mexico (Travassos, 1922; Ruiz, 1947; Wolfgang, 1951; Crites, 1956; Pinto and Gomes, 1980; Martínez, 1986, 1987; Caneda-Guzman, 1997; Gomes *et al*., 2003; Adnet *et al*., 2009; Tantaleán *et al*., 2010; Jiménez *et al*., 2011; Mollericona and Nallar, 2014; Chero *et al*., 2017; Zabott *et al*., 2017; Li, 2019; Cirino *et al*., 2020). *Cruzia tentaculata* is a generalist species with a monoxenous life cycle (Anderson, 2000), although the involvement of gastropod molluscan acting as intermediate hosts has been recently reported (Ramos-de-Souza *et al*., 2021).

Didelphine marsupials have high abundance indices of parasitism by *C. tentaculata* in the Atlantic Forest, which suggests that they play an important role in the maintenance of the parasite metapopulation (Gardner, 1970; Cerqueira, 1985; Cáceres, 2012; Chemisquy and Flores, 2012; Paglia *et al*., 2012; Cole and Viney, 2018; Voss *et al*., 2018). Some processes influencing the population structure of parasites include host mobility, geographical distance between different populations, gene flow and strength of genetic drift (Cole and Viney, 2018). In addition to population genetic structure, parasite geographical variation can also be assessed at the phenotypic level by means of morphological and morphometric studies.

It has been suggested that the use of several host species allows nematodes to cross or colonize a wider range of habitats than would be possible using only a single host, and this tends to promote the genetic flow among parasite populations (Cole and Viney, 2018). The aim was to investigate the existence of cryptic species within *C*. *tentaculata* using the null hypothesis that *C*. *tentaculata* is a single, panmictic population.

We hypothesized that (i) *C*. *tentaculata* represents a panmictic population along the Atlantic Forest, infecting several species of marsupials of the subfamily Didelphinae (host generalist), suggested by the fact that *D*. *aurita* and *D*. *albiventris* have large vagility compared to other small mammals, with the ability to cross degraded areas, exploring anthropized rural and urban environments; and (ii) *C. tentaculata* specimens present morphological variation related to the host localities.

## Materials and methods

### Study areas and sample collection

**Fig. 1.**
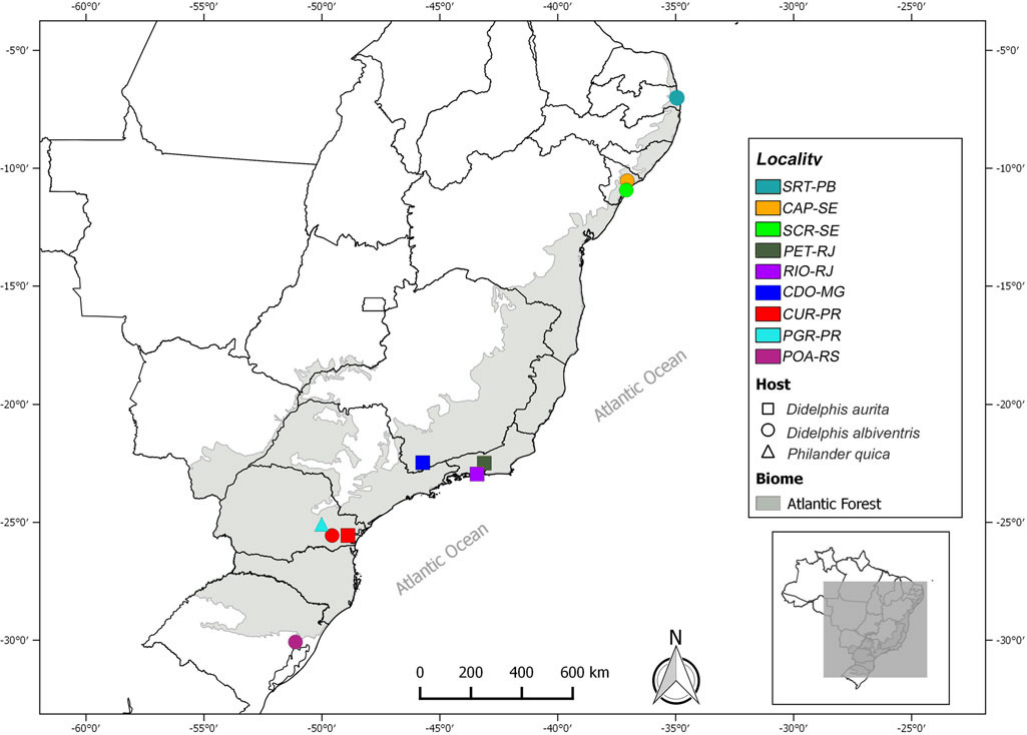
Identification of *Cruzia tentaculata* locality and host species collections carried out along the Atlantic Forest (shaded area in the map).

Specimens of *C*. *tentaculata* were recovered from 3 species of marsupials of the subfamily Didelphinae (*D*. *aurita*, *D*. *albiventris* and *P*. *quica*). These 3 species are the most commonly found and easily captured along the Atlantic Forest on the east coast of South America. Nine different localities were sampled along the Atlantic Forest biome: the municipality of Santa Rita (SRT-PB) in the state of Paraiba, the municipalities of São Cristovão (SCR-SE) and Capela (CAP-SE) in the state of Sergipe, the municipalities of Petrópolis (PET-RJ) and Rio de Janeiro (RIO-RJ) in the state of Rio de Janeiro, the municipality of Conceição dos Ouros (CDO-MG) in the state of Minas Gerais, the municipalities of Curitiba (CUR-PR) and Ponta Grossa (PGR-PR) in the state of Paraná and the municipality of Porto Alegre (POA-RS) in the state of Rio Grande do Sul. The number of helminths studied for each locality was based on the number of hosts collected in each locality and on the parasitic load of each animal to obtain representative samples from each local population (Table 1, Fig. 1).

**Table 1.** Geographic locality, abbreviation, number of hosts, host species, number of specimens of *Cruzia tentaculata* used for genetic and morphometric studies separated by slash and geographic coordinates

Locality	Abbreviation	Number of hosts	Host species	Genetics/morphometry	Geographic coordinates	Locality description
Santa Rita, PB	SRT-PB	4	*D. albiventris*	6, 6, 6, 4/3, 3	07° 05′ 6.41″ S, 34°57′ 0.29″ O	Atlantic Forest domain seasonal eemideciduous forest characterized by a forest formation with typical Atlantic Forest species surrounded by sugarcane cultivation.
Capela, SE	CAP-SE	1	*D. albiventris*	12/6	10° 31′ 44.5″ S, 37° 03′ 35.3″ O	Refúgio da Vida Silvestre Mata do Junco – Atlantic Forest area of seasonal semidual forest. Located in the countryside presented primary and secondary preserved by matrices.
São Cristovão, SE	SCR-SE	2	*D. albiventris*	1, 10/2, 4	37° 06′ 0.90″ S, 10° 55′ 39.8″ O	Campus São Cristóvão, Federal University of Sergipe – an urban interface environment close to disturbed forest fragments in the urban region of the city.
Petrópolis, RJ	PET-RJ	5	*D. aurita*	2, 1, 6, 1, 1/3, 2, 1	22° 29′ 9.60″ S, 43° 07′ 11.3″ O	Serra dos Órgãos National Park – a preserved forest area and one of the most important remnants of Atlantic Forest in Brazil, characterized by a Mountain Atlantic Forest of dense ombrophilous vegetation.
Rio de Janeiro, RJ	RIO-RJ	2	*D. aurita*	11, 10/3, 3	22° 56′ 57.0″ S, 43° 25′ 32.0″ O	FIOCRUZ Atlantic Forest Biological Station – an urban–sylvatic interface environment including peridomicile and disturbed forest areas in the buffer zone of the Pedra Branca State Park, the largest forest reserve in an urban region in the Americas.
Conceição dos Ouros, MG	CDO-MG	2	*D. aurita*	8, 8/3, 3	22° 24′ 42″S; 45° 47′ 24″W	Fazenda Monte Alegre – Atlantic Forest remnant of mixed rainforest, isolated due to degradation promoted by anthropogenic action that transformed the landscape into a matrix of agricultural areas and pasture.
Curitiba, PR	CUR-PR	2, 3	*D. aurita; D. albiventris*	6, 5; 4, 4, 5/2, 6	25° 33′ 19.0″ S, 49° 14′ 0.60″ O	Barigui Park, Acantonamento house, Jardim Botânico Park – an environment characterized by small fragments of altered primary vegetation or secondary vegetation of subtropical forests with araucaria and some preserved areas in and around the city.
Ponta Grossa, PR	PGR/PR	1	*P. quica*	8/6	25° 04′ 45.8″ S 50° 00′47.50″ W	Campos Gerais National Park – Atlantic Forest with remnants of Mixed Ombrophilous Forest and Savannas, the phytocenosis with high species richness, is biogeographically isolated from the other elements of the biome, constituting a possible centre of endemism.
Porto Alegre, RS	POA/RS	15	*D. albiventris*	3, 2, 3, 2, 3, 3, 3, 3, 2, 3, 3, 3 3, 3, 3/3, 3	30° 04′ 17.6″ S, 51° 07′ 28.3″ O	Vila Laranjeiras, Morro do Santana, Morro da Polícia and Campus UFRGS – Peridomicile areas near forest fragments in the urban region of the city.

Marsupials were captured using Tomahawk Live Trap (Hazelhurst, Wisconsin) model 201 traps (16″ × 5″ × 5″). Each trap was baited with a generalist mixture of peanut butter, banana, oats and bacon (nut, fruit, grain and animal protein), preferring odorous items for mammals' attraction. After capture, the marsupials were conducted to a field laboratory, where they were anaesthetized, killed and had their bionomic data and samples taken for different studies.

The marsupials were necropsied, and their organs were removed and placed separately in Petri dishes, washed in saline solution (0.9% NaCl) and dissected with the aid of a stereoscopic microscope. Specimens of *C*. *tentaculata* were recovered through examination of the host caecum and large intestine. The helminths were washed in saline solution and stored in 70% ethanol for further morphological, morphometric and molecular studies.

For morphological characterization and morphometric description, the specimens were clarified in 25% glycerinated alcohol, mounted on a slide under a coverslip, identified and measured with the aid of camera lucida attached to a Nikon Eclipse E200 MV R microscope. The identification of species by morphology and morphometry was performed using articles describing species of the genus *Cruzia* and taxonomic keys according to Vieira *et al*. (2020). The morphometric characteristics analysed for males and females were the length and width of the oral capsule, length and width of the body, length and width of the oesophagus, length and width of the bulb, distance from the nerve ring and excretory pore with respect to the anterior extremity and length of the intestinal diverticulum. Only for males were the following characteristics measured: length of the spicules, length of the gubernaculum and distance from the cloaca to the posterior extremity. For females, the following characteristics were also measured: the distance from the vulva to the anterior extremity and from the anus to the posterior extremity. The specimens used in the morphometric analyses were not the same as those used in the molecular analyses and in the morphological analyses by scanning electron microscopy (SEM), so it was not possible to correlate them. For SEM analyses, the fixed nematodes were washed in 70% ethanol, dehydrated in a graded ethanol series (70–100%), critical point, dried in CO_2_, free-hand fractured, mounted on metallic stubs and coated with gold. The samples were analysed using a scanning electron microscope Jeol JSM 6390LV on the Electron Microscopy Platform Rudolf Barth, Instituto Oswaldo Cruz, Fiocruz.

### Discriminant analysis of principal components

A discriminant analysis of principal components (DAPC) (Jombart *et al*., 2010) was performed to compare *C. tentaculata* specimen measurements among localities (PET-RJ, RIO-RJ, CUR-PR, PGR-PR, POA-RS, SCR-SE, CAP-SE, CDO-MG) and among host species (*D. albiventris*, *D. aurita* and *P. quica*). In this analysis, the same morphometric characteristics described above were used. The DAPC describes variations between defined groups, selecting principal components (PCs) that explain the greatest variation between groups while minimizing the variation within each group (Jombart and Collins, 2015). To identify the optimal number of PCs to be retained by DAPC and select the components associated with the lowest root mean-squared error, we used cross-validation optimization (Jombart and Collins, 2015). We determined the percentage of *C. tentaculata* specimens correctly classified within their original group. Finally, we tested the significance of the differences found within each group using Wilks' Lambda statistic.

DAPC analysis was performed for both male and female specimens of *C. tentaculata*, using only those specimens with sufficient information on morphometric measurements within each category among the groups represented by locality and host species. For male specimens, the locality of CUR-PR was removed due to its small sample size and the absence of morphometric measurements resulting from the preservation status of the material. Similarly, for female specimens, the locality of PGR-PR, POA-RS and SRT-PB and the species *P. quica* were removed, also due to small sample size or due to poor preservation of the material, preventing the visualizations of some structures. DAPC was performed using the package ‘adegenet’ (Jombart, 2008), and one-way MANOVA – Wilks' lambda was performed using the package ‘rrcov’ (Todorov and Filzmoser, 2009), both in the R software environment version 4.1.2 (R Core Team, 2021).

### Molecular and phylogenetic analyses

For molecular characterization, total genomic DNA of *C*. *tentaculata* specimens was isolated using the extraction kit NucleoSpin Tissue (Macherey-Nagel, Düren, Germany) according to the manufacturer's instructions and stored at −20°C until use. Partial cytochrome c oxidase subunit 1 (MT-CO1) mitochondrial gene fragments were amplified by polymerase chain reaction (PCR) using the primer cocktail described by Prosser *et al*. (2013). Amplifications were performed at a final volume of 25 *μ*L for each sample, comprising 12.5 *μ*L of PCR Master Mix (Taq DNA polymerase 50 units mL^−1^, dATP 400 *μ*m, dGTP 400 *μ*m, dCTP 400 *μ*m, dTTP 400 *μ*m, MgCl2 3 mm) (Promega, Madison, Wisconsin, United States); 10.5 *μ*L of ultrapure water for molecular biology, 0.5 *μ*L of sense (primers Nem_F1, Nem_F2 and Nem_F3) and of anti-sense (primers Nem_R1, Nem_R2 and Nem_R3) oligonucleotide cocktail (10 *μ*m); and 1.0 *μ*L of genomic DNA (~1–4 ng *μ*L^−1^). We used a Veriti Thermal Cycler (Life Technologies) thermocycler under the following thermal cycling conditions: initial denaturation at 94°C for 1 min; 5 cycles of denaturation at 94°C for 40 s, hybridization at 45°C for 40 s, and extension at 72°C for 1 min; followed by 35 cycles of denaturation at 94°C for 40 s, hybridization at 51°C for 40 s, and extension at 72°C for 1 min; and a final extension at 72°C for 5 min (Prosser *et al*., 2013).

The resulting amplicons were visualized using 1.5% agarose gel electrophoresis with GelRed Nucleic Acid Stain (Biotium, Hayward, California, USA) on an ultraviolet light transilluminator. Successfully amplified PCR products were purified using the Illustra GFX PCR DNA and Gel Band kit (GE Healthcare, Chicago, Illinois, USA) according to the manufacturer's instructions and sequenced using the BigDye Terminator v3.1 Cycle Sequencing kit (Applied Biosystems, USA). The reactions were prepared at a final volume of 10 *μ*L for each sample using the primer cocktail described by Prosser *et al*. (2013). Sequencing reactions were performed on a Veriti Thermal Cycle (Life Technologies) thermocycler under the following cycling conditions: denaturation at 94°C for 10 s, hybridization at 50°C for 5 s and extension at 60°C for 4 min, followed by 39 repetitions of this cycle. Subsequently, cycle-sequencing product precipitation, formamide resuspension and analysis using the ABI3730xl DNA Analyzer (Applied Biosystems) were conducted at the Capillary Sequencing (SANGER) Platform of the Fiocruz Technological Platforms Network at the Oswaldo Cruz Institute (https://plataformas.fiocruz.br/unidades/RPT01A).

After sequencing, the forward and reverse sequences obtained were assembled into contigs and edited for ambiguities using the Geneious R9 software package (Kearse *et al*., 2012). To build our data set, we include sequences of species representatives of the order Ascaridida Skrjabin & Shulz, 1940. As an outgroup, we added sequences of *Ascaris lumbricoides* (Linnaeus, 1758); *Baylisascaris schroederi* (Mclntosh, 1939); *Parascaris equorum* (Goeze, 1782); *Pseudoterranova krabbei* Paggi, Mattiucci, Gibson, Berland, Nascetti, Cianchi and Bullini, 2000; *Toxascaris leonina* (Von Linstow, 1902); and *Toxocara cati* (Schrank, 1788) based on phylogenetic proximity to the Kathlaniidae family (Table 2). Then, they were aligned by multiple alignment using the MUSCLE algorithms (Edgar, 2004) on the Translator X server (Abascal *et al*., 2010), which employs amino acid translations to align protein-coding nucleotide sequences.

**Table 2.** Species, GenBank accession number, geographic locality and host of Ascaridida GenBank sequences used in this study

Species	Access number	Locality	Hosts	Reference
*Cruzia tentaculata*	OM670268 – OM670326	Brazil	*Didelphis aurita*	Present study
*Cruzia tentaculata*	OM670327 – OM670426	Brazil	*Didelphis albiventris*	Present study
*Cruzia tentaculata*	OM670427 – OM670434	Brazil	*Philander quica*	Present study
*Cruzia tentaculata*	MN842777	Brazil	*Achatina fulica*	Ramos-de-Souza *et al*. (2021)
*Cruzia tentaculata*	MN842778	Brazil	*Achatina fulica*	Ramos-de-Souza *et al*. (2021)
*Ascaris lumbricoides*	JN801161	South Korea	Human	Park *et al*. (2011)
*Baylisascaris schroederi*	HQ671081	China	Giant panda	Xie *et al*. (2011)
*Parascaris equorum*	MF678786	China	Horse	Gao *et al*. (2019)
*Pseudoterranova krabbei*	NC_031646	Norway	Grey seal	Liu *et al.* (2016)
*Toxascaris leonina*	KC902750	Australia	Dog	Liu *et al*. (2014)
*Toxocara cati*	AM411622	China	–	Li *et al*. (2008)

Phylogenetic analyses were performed using the maximum likelihood (ML) and Bayesian inference (BI) methods. Phylogenetic reconstructions by ML were conducted using the online web server PhyML 3.0 (Guindon *et al*., 2010). The selection of the nucleotide substitution model was performed with SMS (Smart Model Selection) (Lefort *et al*., 2017) in PhyML using the Akaike information criterion (AIC). The nucleotide replacement model selected by the AIC in PhyML SMS was GTR + R. Statistical support to evaluate the reliability of the branches was obtained by bootstrap (BP) with 1000 pseudoreplicas and the approximate likelihood-ratio test for branches (aLRT). BI analyses were conducted using the program MrBayes 3.2.6 (Ronquist *et al*., 2012), with independent GTR + I + G nucleotide substitution models for each codon position and unlinking base frequencies and parameters. Sampling was performed every 100 generations for 10 000 000 generations, with 4 simultaneous chains, in 2 runs, by MCMC. The robustness of the nodes was evaluated by Bayesian posterior probabilities (BPP), calculated after the removal of a burn-in fraction of 25%. To evaluate the adequacy of our sampling, we used the program Tracer 1.6 (Rambaut *et al*., 2014) to calculate the effective sample size (ESS) of each parameter. MrBayes was run on XSEDE using the CIPRES Science Gateway (Miller *et al*., 2010).

### Population genetic analyses

To determine whether the genes were under selection, we performed neutrality tests to identify whether there was a deviation of the balance. Tajima's *D* (Tajima, 1989) and Fu's *F*s (Fu, 1997) neutrality tests were performed for each of the previously determined groups (i.e. based on hosts and locality) in the program Arlequin 3.5.2.2 (Excoffier and Lischer, 2010). To identify population structuring, fixation indices (Fst) (Wright, 1978) were calculated to evaluate genetic differentiation between localities. Molecular variance analysis (AMOVA) (Excoffier *et al*., 1992) was used to analyse the genetic variability between and within groups previously defined using the program Arlequin, and we also performed a Bayesian clustering analysis implemented in the program Bayesian Analysis of Population Structure (BAPS). Haplotype networks were calculated for the *C*. *tentaculata* sequences using the median joining (MJ) method (Bandelt *et al*., 1999) in the program PopART 1.7 (Leigh and Bryant, 2015) to assess the distribution and sharing of haplotypes among populations.

The correlation between the genetic distances of all specimens of *C*. *tentaculata* and the geographical distances, considering all the localities studied, was investigated using the Mantel correlation test (Legendre and Legendre, 2012). A genetic distance matrix was calculated in the ‘ape’ package (Paradis and Schliep, 2019), the geographic distance matrix was calculated in the ‘fields’ package (Nychka *et al*., 2017), and the Mantel correlation test was performed in the ‘vegan’ package (Oksanen *et al*., 2020), all using the software environment R 4.1.0 (R Core Team, 2021).

## Results

### Morphological analysis by light, scanning electron microscopy and morphometric analysis

**Fig. 2.**
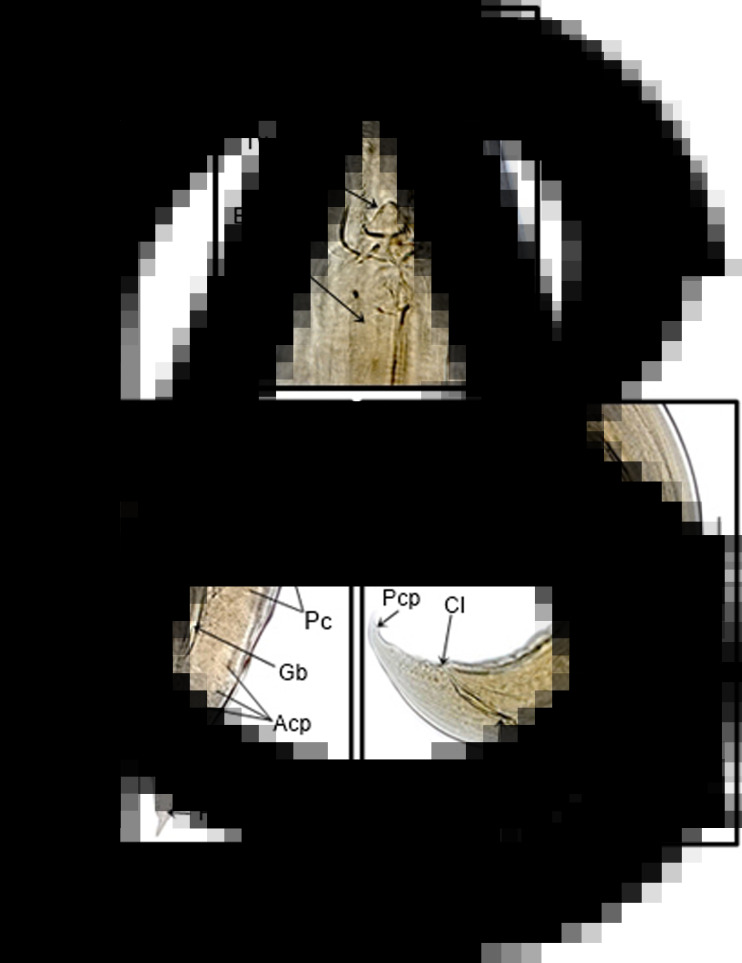
Light microscopy photographs of an adult male specimen of *Cruzia tentaculata* from the SRT-PB locality. (A) Details of the anterior region showing the lips (Lp), cuticular lamellae (Cl), tricuspid valve (Tv) and muscular part of the esophagus (Es) (bar: 50 *μ*m). (B) Ventral view of the posterior region of the male showing spicules (Sp), gubernaculum (Gb), small caudal wing (Cw), 2 pairs of precloacal papillae (Pc), 3 pairs of ad-cloacal papillae (Acp) and 1 postcloacal papilla (Pcp) (bar: 50 *μ*m). (C) Lateral view of the posterior region of the male showing spicules (Sp), gubernaculum (Gb), cloaca (Cl) and 1 postcloacal papilla (Pcp) (bar: 100 *μ*m).

The measured male specimens of *C*. *tentaculata* have a body length ranging from 8.52 to 13.63 mm and females ranging from 10.52 to 16.13 mm. The anterior opening of the nematodes in both sexes is surrounded by 3 subtriangular lips, a dorsal lip with a pair of papillae on each side and 2 latero-ventral lips with a papilla and amphids on each side. Lips have 2 small teeth located on the inner margin. The pharynx has 3 rows each with 2 faces provided of 12 longitudinal pairs of cuticular lamellae and a tricuspid valve at the base (Fig. 2A). The oesophagus is cylindrical and long, divided into muscle and glandular portions, presenting a prebulbar dilation followed by a well-developed bulb containing 1 valve and accompanied by bulb valves at the base. The nerve ring is in the anterior oesophageal region, and the excretory pore is in the posterior third of the oesophageal region. The intestinal diverticulum is directed to the anterior region, exceeding the threshold of the oesophageal bulb. Males were curved ventrally in the posterior region, and the cloaca opening was at the caudal end. The caudal ventral region shows 10 pairs of bud-like papillae, 3 precloacal pairs, 3 ad-cloacal pairs and 4 postcloacal pairs, as well as a single papilla at the anterior edge of the cloacal opening. The spicules are subequal and alates, and the gubernaculum is triangular and has a slender caudal alae (Fig. 2B and C). The females are didelphic and amphidelphic, with the vulva in the preequatorial region and the tail ending in a thin tip.

**Fig. 3.**
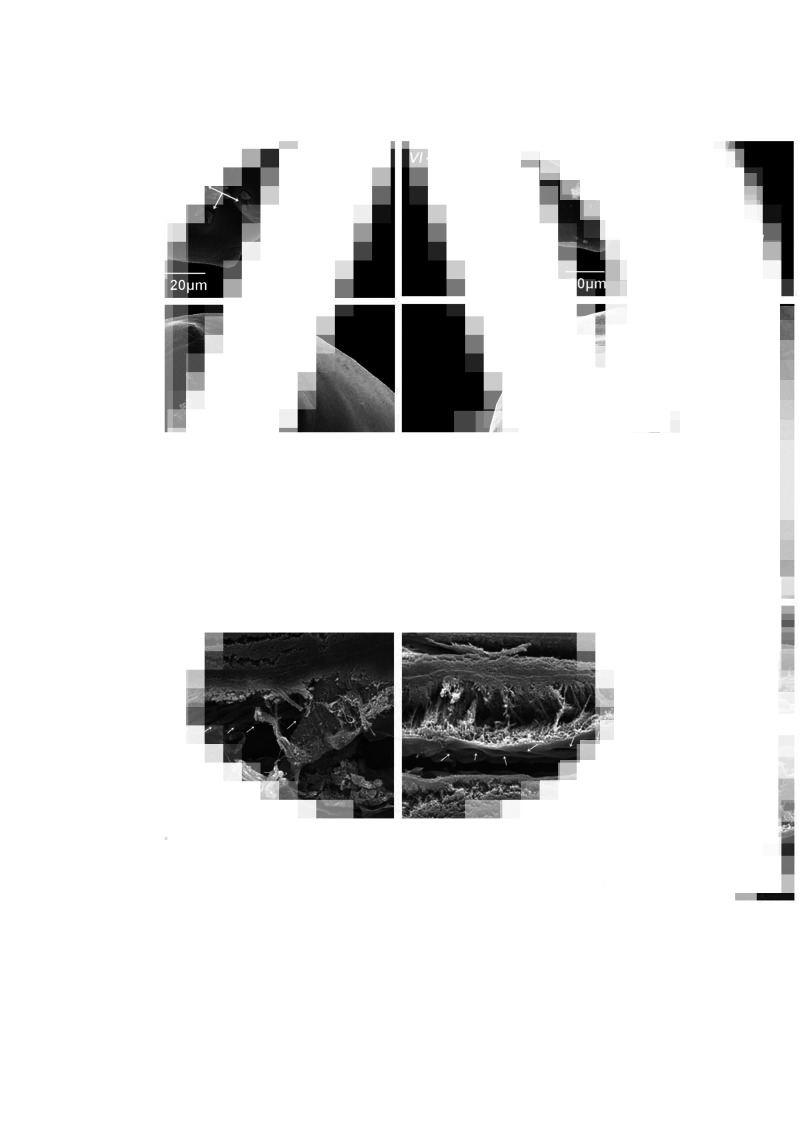
Scanning electron microscopy image of the anterior region of adults of *Cruzia tentaculata*. A–D: anterior extremity with 3 lips with a pair of small teeth located on the inner margins (triple arrow), dorsal lips (Dl) and latero-ventral (Vl). E and F: Anterior region of the nematodes fractured longitudinally, showing the internal surface of the pharynx ornamented with a row of pharyngeal lamellae with 12 pairs of cuticular lamellae (small arrows).

SEM analyses were performed to observe the cuticular lamellae of *C*. *tentaculata* specimens. The preparation required a longitudinal fracture of the oral capsule of some specimens. We observed 3 rows with 2 longitudinal facets with 12 pairs of well-developed cuticular lamellae (Fig. 3A and B).

### Discriminant analysis of principal components

MANOVA of morphometric variables showed that female specimens of *C*. *tentaculata* were significantly different among localities and host species (Table 3); however, this difference was more pronounced for the former, given the lower Wilks' lambda value for localities (Wilks' lambda < 0.01; *P* = <0.01) in relation to host species (Wilks' lambda = 0.07; *P* = <0.01) (Table 3). Males were significantly different among localities (Wilks' lambda < 0.01; *P* < 0.01) and among hosts (Wilks' lambda = 0.02; *P* = 0.05), although to a lesser extent in the latter (Table 3).

**Table 3.** Morphometric divergence of *Cruzia tentaculata* male and female specimens

*Cruzia tentaculata*	Group	Wilks' lambda	Chi^2^-value	d.f.	*P* value
Male specimens	Host species	0.02	43.36	30	0.05
	Locality	<0.01	418.95	90	<0.01
Female specimens	Host species	0.07	28.67	13	<0.01
	Locality	<0.01	107.9	65	<0.01

d.f., degrees of freedom.

Comparison between groups (host species and locality) based on values of the classical and robust one-way MANOVA – Wilks' lambda.

In the DAPC for female parasites, 4 principal components (PCs) were retained to discriminate host species groups, and 2 PCs were retained to discriminate locality groups. For males, 2 PCs were retained to discriminate both host species and locality groups. In each of these DAPCs, the principal components retained explained approximately > 98% of the total variance. The proportion of female specimens correctly classified to their original groups was approximately 50% for localities and 86% for host species. For males, the proportion of specimens correctly classified to their original groups was approximately 57% for localities and 75% for host species.

**Fig. 4.**
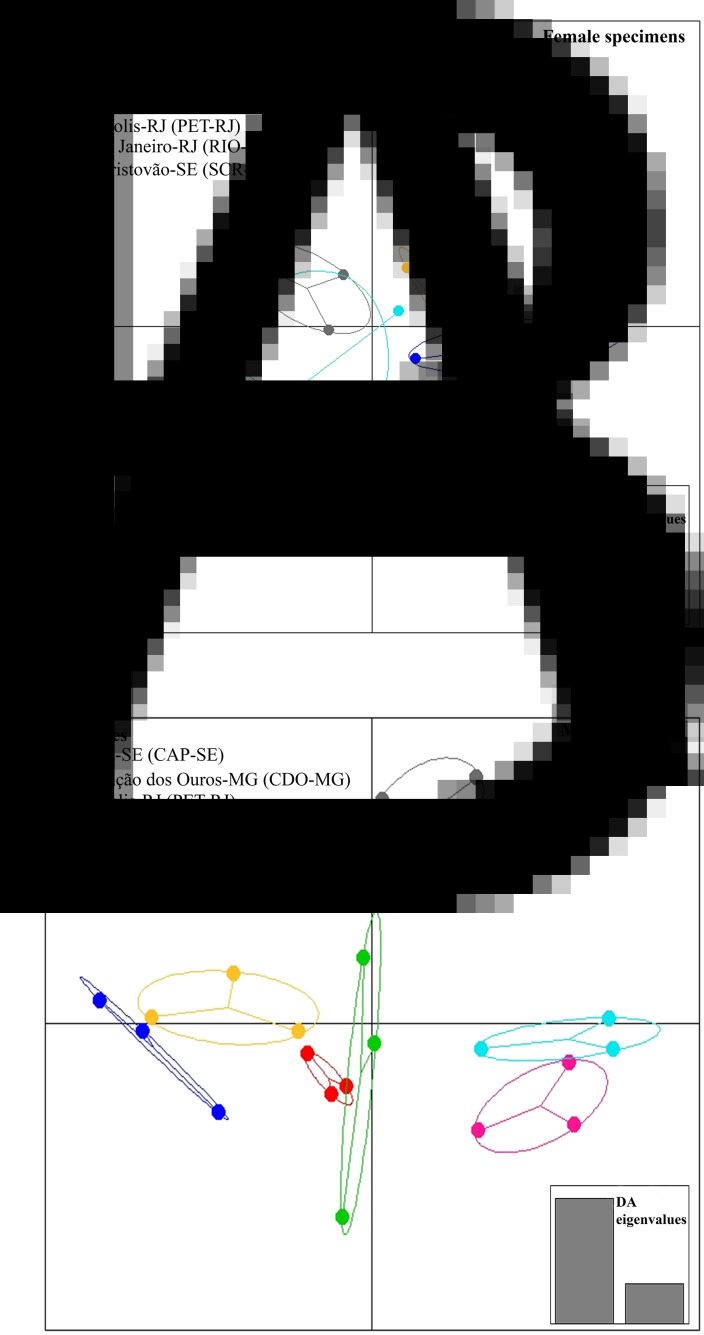
Population clusters for female (A) and male (B) *Cruzia tentaculata* specimens, based on DAPC along with discriminant analysis (DA) eigenvalues, showing morphometric variations between localities.

**Fig. 5.**
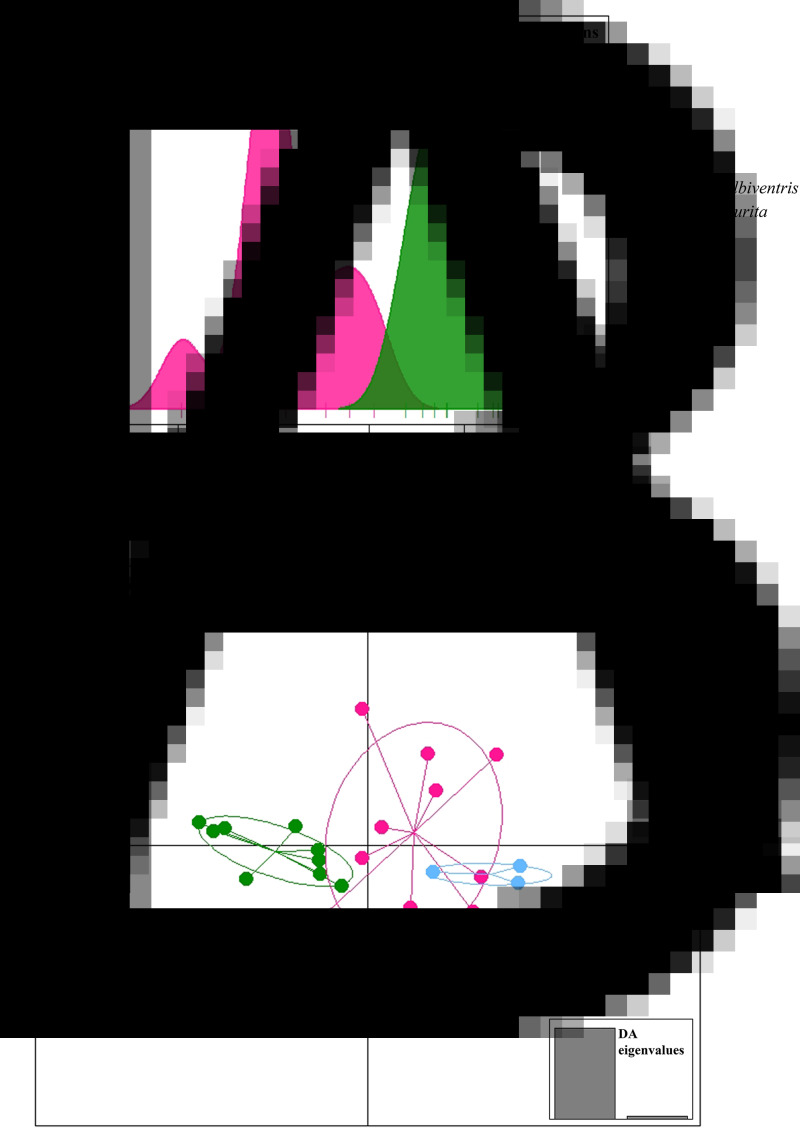
Population clusters for female (A) and male (B) *Cruzia tentaculata* specimens, based on DAPC along with discriminant analysis (DA) eigenvalues, showing morphometric variations between host species.

When analysing the differences between localities, DAPC identified 2 sets of localities for females, 1 from the southeast region (RIO-RJ, PET-RJ and CDO-MG) and the other formed by the localities of the northeast region (CAP-SE and SCR-SE) and (CUR-PR) southern region (Fig. 4A). For males, 2 sets of localities were formed, 1 formed by a locality of the northeast region (SCR-SE) and a locality of the southern region (PGR-PR) and another formed by a locality of the southeast region (RIO-RJ, PET-RJ and CDO-MG) and a locality of the southern region (POA-RS) (Fig. 4B). The locality CAP-SE was isolated in the DAPC plot (Fig. 4B), indicating that the male specimens studied in this locality had unique morphometric characteristics (e.g. higher mean values of morphometric variables or lower variation of these variables among specimens in this locality compared to the others). In turn, as pointed out by MANOVA, differences between host species in DAPC were less evident than between localities, since total isolation was not observed between parasites across host species (Fig. 5A and B), which may indicate a greater degree of morphometric similarity between specimens of *C. tentaculata* occurring in different host species than among some localities.

**Fig. 6.**
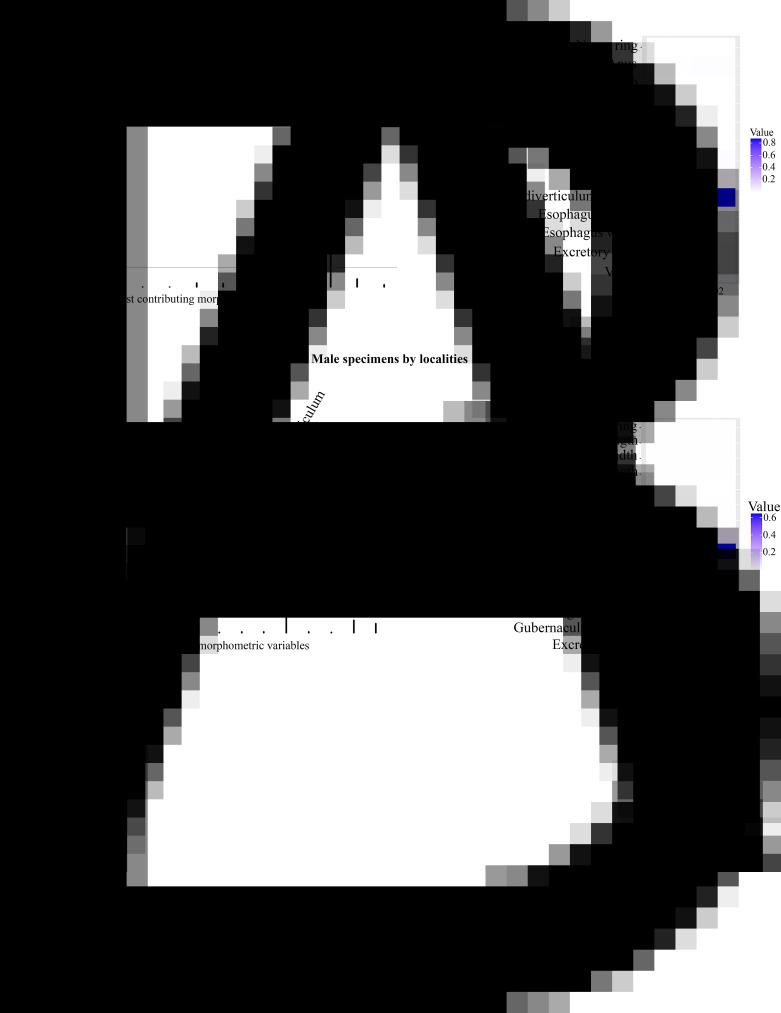
Morphometric variable contributions to *Cruzia tentaculata* specimens among localities. (A) Morphometric variable contributions to female *Cruzia tentaculata* specimens; (B) Morphometric variable contributions to male *Cruzia tentaculata* specimens.

**Fig. 7.**
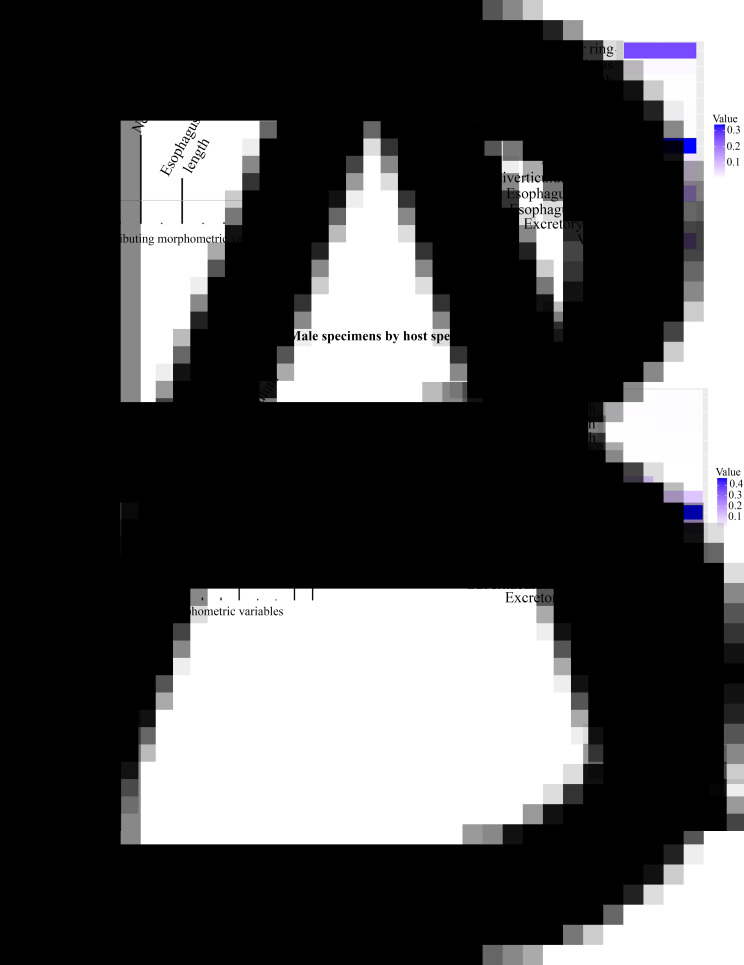
Morphometric variable contributions to *Cruzia tentaculata* specimens among host species. (A) Morphometric variable contributions to female *Cruzia tentaculata* specimens; (B) Morphometric variable contributions to male *Cruzia tentaculata* specimens.

The variables that best discriminated parasites between the localities for female specimens were the length of the intestinal diverticulum and the body length (Fig. 6A). For males, those variables were the length of the intestinal diverticulum, the excretory pore and the length of the oesophagus (Fig. 6B). Regarding host species, the variables that best discriminated female parasites were body length, nerve ring, oesophagus length and vulva position (Fig. 7A). For males, those variables were the length of the intestinal diverticulum, the length of the excretory pore, the length of the spicules (short and long), and the body width (Fig. 7B).

### Genetic diversity indices and population structure

In the present study, among 164 partial sequences of the MT-CO1 gene of *C*. *tentaculata* studied, 144 haplotypes with 161 polymorphic sites were found, and the mean nucleotide diversity and haplotypic diversity were 0.03480 and 0.998, respectively. To estimate the genetic diversity indices, the *C*. *tentaculata* sequences were organized into groups according to the host species and localities studied (Table 4).

**Table 4.** *Cruzia tentaculata* specimens partial MT-CO1 gene sequences genetic diversity based on host species and locality

Host	N	H	S	Hd	*π*
*D. aurita*	57	57	115	1.000	0.01983
*D*. *albiventris*	99	80	129	0.995	0.04129
*P*. *quica*	8	8	32	1.000	0.01830
Locality					
SRT-PB	21	19	61	0.990	0.02042
PET-RJ	10	10	37	1.000	0.01421
RIO-RJ	20	20	66	1.000	0.01993
CUR-PR	24	22	69	0.993	0.02037
PGR-PR	8	8	32	1.000	0.01830
SCR-SE	11	8	65	0.927	0.04308
CAP-SE	12	10	44	0.970	0.02005
CDO-MG	16	16	55	1.000	0.01598
POA-RS	42	32	59	0.985	0.01702

N, sample size; H, number of haplotypes; S, number of polymorphic sites; Hd, haplotype diversity; *π*, nucleotide diversity.

Three matrices were constructed. The first was generated to calculate genetic diversity indices, AMOVA, Fst, Tajima's *D* and Fu's *F*s neutrality tests, and the Mantel test, containing only the sequences of *C*. *tentaculata* generated in the present study, totalling 164 sequences with 693 bp. The second was composed of sequences obtained in the present study and 2 sequences of *C*. *tentaculata* species from GenBank, totalling 166 sequences with 693 bp, used to calculate haplotype networks and the Bayesian clustering analysis. The third matrix was composed of a representative of each haplotype obtained in the present study, 2 sequences of species of *C*. *tentaculata* from GenBank as an ingroup, and 6 sequences from GenBank of the order Ascaridida as an outgroup, resulting in 152 sequences, each with 693 base pairs (bp) used for phylogenetic inferences.

**Fig. 8.**
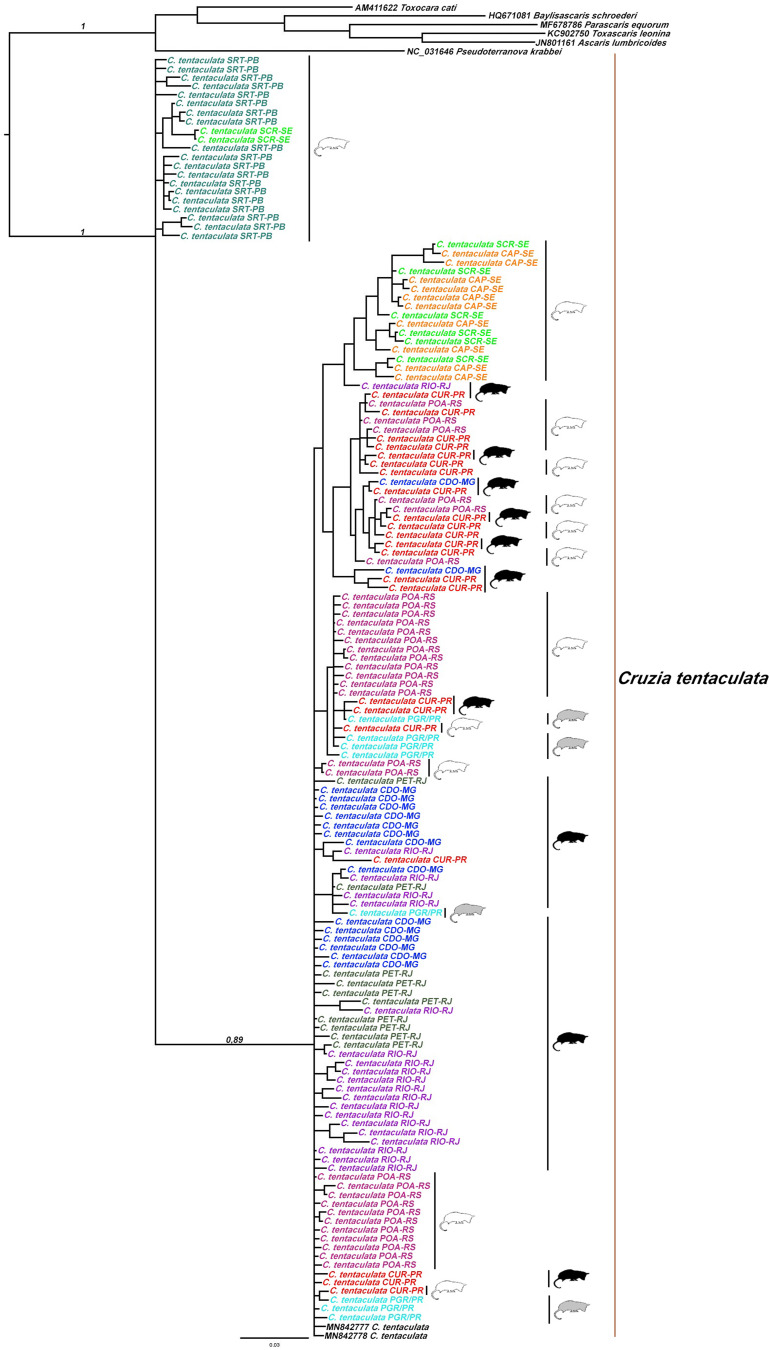
Phylogenetic reconstruction by Bayesian inference (BI) based on partial MT-CO1 gene sequences (693 bp) of 164 specimens of *Cruzia tentaculata*, parasites of *Didelphis albiventris* (white), *Didelphis aurita* (black), and *Philander quica* (grey) from the Atlantic Forest biome, 2 sequences of species of *Cruzia tentaculata* from GenBank and 6 sequences of other species belonging to the order Ascaridida as an outgroup. Values at nodes are the Bayesian posterior probabilities (BPP). Colours represent each locality in relation to the network of haplotypes.

In the ML analysis, the nucleotide replacement model selected by the AIC in PhyML SMS was GTR + R, with an estimated invariable site proportion of 0.000, estimated gamma parameter of 1.000 and variation of rates with 4 categories. The most likely tree presented an LnL of −4970.750524. The estimated base frequencies were A = 0.15645, C = 0.06752, G = 0.26508, and T = 0.51095. For BI, the estimated mean marginal probability was −4982.4043, and the median was −4982.079. The ESSs for all parameters were above 100 samples and were effectively independent, indicating the robustness of our sampling. Phylogenetic reconstructions by ML and BI resulted in equal topologies. Specimens of *C*. *tentaculata* formed a monophyletic group in both phylogenies (aLRT = 0.99; BP-MV = 0.99; BPP = 1.00). In the ML (Supplementary material) and BI (Fig. 8), the group of *C*. *tentaculata* was composed of parasites from *D*. *albiventris* in the state of Paraíba (SRT), Sergipe (CAP and SCR), Paraná (CUR), Rio Grande do Sul (POA), by parasites from *D*. *aurita* in the states of Rio de Janeiro (RIO and PET), Paraná (CUR) and Minas Gerais (CDO) and from *P*. *quica* in the state of Paraná (PGR).

**Fig. 9.**
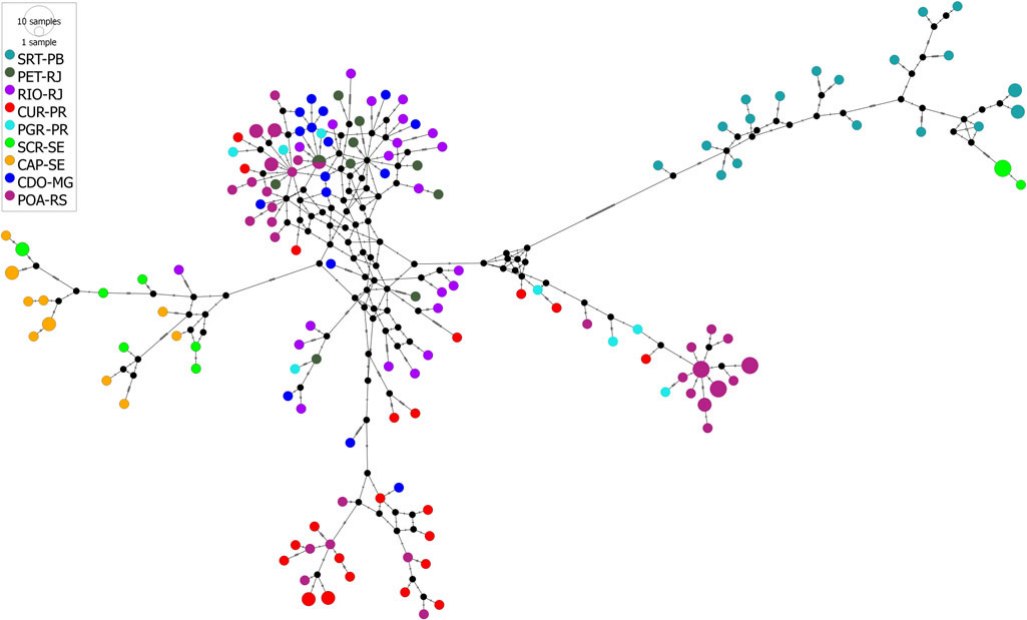
Median-joining haplotype network based on partial MT-CO1 gene sequences (693 bp) of *Cruzia tentaculata*, a parasite of the caecum and large intestine of *Didelphis albiventris*, *Didelphis aurita* and *Philander quica*, from this study and 2 sequences of species of *Cruzia tentaculata* from GenBank. The size of the circles represents the frequency of haplotypes. The colours of the circles represent the occurrence locality of each haplotype. In the network, 2 groups were observed: group I (SRT-PB and SCR-SE) and group II (PET-RJ, RIO-RJ, POA-RS, CUR-PR, PGR-PR, SCR-SE, CAP-SE and CDO-MG).

**Fig. 10.**
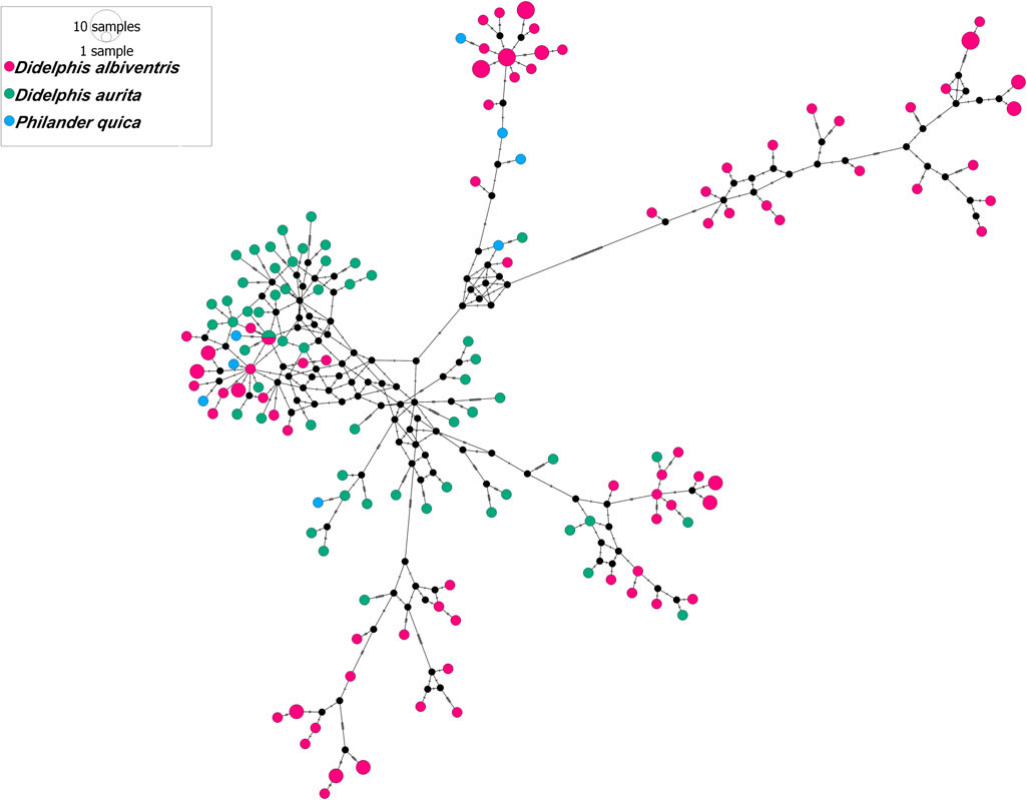
Median-joining haplotype network based on partial MT-CO1 gene sequences (693 bp) of *Cruzia tentaculata*, a parasite of the caecum and large intestine of *Didelphis albiventris*, *Didelphis aurita* and *Philander quica*, from this study and 2 sequences of species of *Cruzia tentaculata* from GenBank. The size of the circles represents the frequency of haplotypes. The colours of the circles represent the occurrence hosts of each haplotype.

The haplotype network suggested separation of 2 groups: SRT-PB and SCR-SE (group I) and PET-RJ, RIO-RJ, POA-RS, CUR-PR, PGR-PR, SCR-SE, CAP-SE and CDO-MG (group II), separated by a genetic distance of 32 mutational steps. The sharing of haplotypes between localities and host species was observed. Haplotype H-5 was shared between the POA-RS and PET-RJ localities (Fig. 9). Haplotype H-5 was shared between *D*. *albiventris* and *D*. *aurita*. Interestingly, in the host haplotype network, there were 3 groups of haplotypes for *D. albiventris* at the periphery of the haplotypes of *D. aurita* (Fig. 10).

**Fig. 11.**
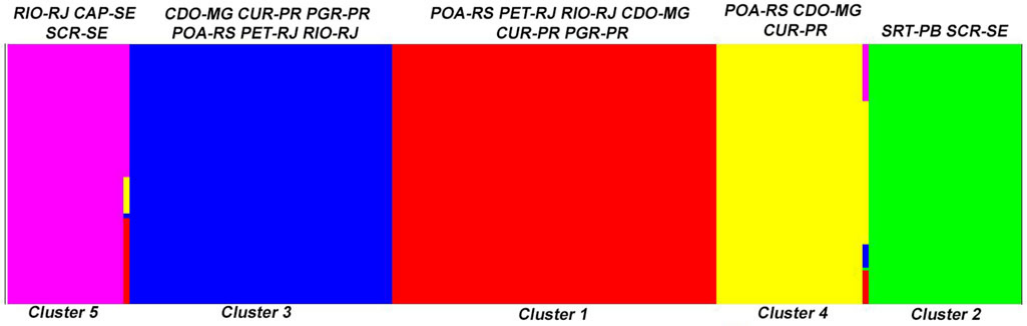
Population structure of *Cruzia tentaculata* deduced by Bayesian analysis of population structure. Structural plot of 166 specimens of *Cruzia tentaculata* revealing 5 different major populations (clusters 1–5). The locations of each cluster are represented in the graph.

Regarding hosts, the AMOVA indicated higher genetic variance within host (88.85%) species than between hosts. There was a higher genetic variation between localities (52.82%) than within localities (Table 5). The value of Fst for host species was significant when compared between *D*. *aurita* and *D*. *albiventrtis* (0. 002). For localities, the Fst values were significant between CDO-MG and SCR-SE, CDO-MG and CAP-SE, SCR-SE and POA-RS, SCR-SE and CUR-PR, SCR-SE and RIO-RJ, SCR-SE and CAP-SE, SCR-SE and SRT-PB, POA-RS and CUR-PR, POA-RS and CAP-SE, POA-RS and SRT-PB, CUR-PR and CAP-SE, CUR-PR and SRT-PB, RIO-RJ and CAP-SE and CAP-SE and SRT-PB (Table 6). The neutrality tests Tajima's *D* and Fu's *F*s were determined for each group. For the comparisons formed by the host species, *D* values were significant only for the group of *C*. *tentaculata* parasites from *D*. *aurita*, while *F*s values were significant for both *D*. *aurita* and *D*. *albiventris*. Considering the localities, the *D* values were not significant. The *F*s values were significant for SRT-PB, PET-RJ, RIO-RJ, CUR-PR, CDO-MG and POA-RS (Table 7). According to the population structure using BAPS, specimens of *C*. *tentaculata* were distributed in 5 clusters (Fig. 11).

**Table 5.** *Cruzia tentaculata* specimens partial MT-CO1 gene sequence genetic variability by AMOVA based on host species and localities

Source of variation	d.f.	Percentage of variation
Host		
Among hosts	2	11.15
Within hosts	161	88.85
Total	163	
Locality		
Among locality	8	51.58
Within locality	155	48.42
Total	163	

d.f., degrees of freedom.

**Table 6.** *Cruzia tentaculata* specimens partial MT-CO1 gene sequences genetic variance estimation by Fst based on collection locality

	CDO-MG	PGR-PR	SCR-SE	POA-RS	CUR-PR	PET-RJ	RIO-RJ	CAP-SE	SRT-PB
CDO-MG									
PGR-PR	0.000								
SCR-SE	**0**.**035**	0.037							
POA-RS	0.007	0.008	**0**.**041**						
CUR-PR	0.003	0.003	**0**.**037**	**0**.**011**					
PET-RJ	0.000	0.000	0.036	0.005	0.003				
RIO-RJ	0.000	0.000	**0**.**034**	0.007	0.003	0.000			
CAP-SE	**0**.**014**	0.015	**0**.**051**	**0**.**022**	**0**.**018**	0.015	**0.014**		
SRT-PB	0.004	0.005	**0**.**039**	**0**.**012**	**0**.**008**	0.005	0.004	**0.019**	

Significant values are in bold (*P* < 0.05).

**Table 7.** Neutrality tests (Tajima's *D* and Fu's *F*'s), with the respective *P* values, of *Cruzia tentaculata* specimens partial MT-CO1 gene sequences grouped into host species and locality

Hosts	Tajima's *D*	*P*	Fu's *F*	*P*
*D. aurita*	**−1.57715**	0.02700	**−24.32267**	0.00000
*D. albiventris*	0.48564	0.74400	**−23.95827**	0.00000
*P. quica*	0.14506	0.59100	−1.70085	0.12000
Locality				
SRT-PB	−0.66456	0.29100	**−5.74522**	0.02200
PET-RJ	−1.19738	0.11500	**−3.52756**	0.03900
RIO-RJ	−1.04711	0.13700	**−9.82066**	0.00200
CUR-PR	−0.92703	0.18300	**−8.05837**	0.00500
PGR-PR	0.14506	0.55200	−1.70085	0.14500
SCR-SE	1.64155	0.97100	3.02980	0.89700
CAP-SE	−0.21211	0.41500	−0.62131	0.34800
CDO-MG	−1.41008	0.07200	**−7.75106**	0.00300
POA-RS	−0.49925	0.35500	**−12.44708**	0.00100

Significant values are in bold (*P* < 0.05).

**Fig. 12.**
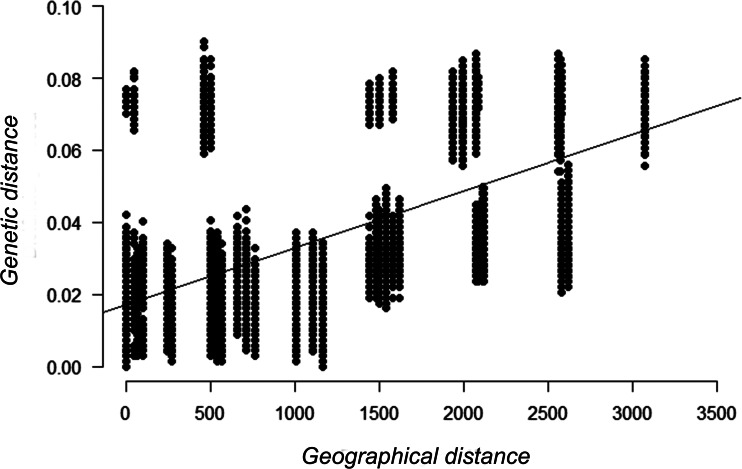
Mantel test result showing the correlation between genetic and geographic distances in kilometres (*r*: 0.6764 *P* = 0.001).

The Mantel test indicated that approximately 68% of the genetic differentiation was explained by the spatial distance between the localities (correlation coefficient, *r*: 0.6764 *P* = 0.001) (Table 8, Fig. 12).

**Table 8.** Geographic distance matrix in kilometres

	SCR-SE	CAP-SE	CDO-MG	PGR-PR	PET-RJ	RIO-RJ	SRT-PB	CUR-PR
SCR-SE								
CAP-SE	35							
CDO-MG	1.357	1.384						
PGR-PR	1.829	1.852	515					
PET-RJ	1.188	1.219	290	792				
RIO-RJ	1.238	1.269	259	751	50			
SRT-PB	425	393	1.771	2.226	1.613	1.662		
CUR-PR	1.792	1.817	454	93	720	676	2.196	
POA-RS	2.173	2.198	821	373	**1.059**	1.010	2.579	384

Shared haplotype: H-5 (POA-RS and PET-RJ).

## Discussion

### Morphological diversity and morphometric comparison between *Cruzia tentaculata*

Morphological analyses performed by SEM and light microscopy confirmed the identification of the studied specimens as *C*. *tentaculata*, agreeing with the taxonomic key (Vieira *et al*., 2020) and articles discussing the species (Travassos, 1922; Adnet *et al*., 2009). Most studies of morphological descriptions of the species do not examine the number of cuticular lamellae. According to Ruiz (1947) and Adnet *et al*. (2009), *C*. *tentaculata* presents only 10 pairs of tooth-like structures. Crites (1956) examined 6 male specimens of *C*. *tentaculata* parasitic in *D. aurita* collected in Brazil and reported that specimens of *C*. *tentaculata* have 8–14, generally 10, cuticular lamellae. Comparing our samples with 11 specimens of *C*. *tentaculata* from the Helminthological collection of the Oswaldo Cruz Institute (CHIOC) (Fiocruz-CHIOC 1556 – parasitic in *Didelphis* sp. identified by Travassos; Fiocruz-CHIOC 33678 – parasitic in *Didelphis aurita* identified by Vicente & Cruz, 1997; and Fiocruz-CHIOC 35624 – parasite of *Didelphis aurita* identified by Adnet *et al*., 2009), all observed specimens presented 12 pairs of cuticular lamellae similar to those observed in the specimens of the present study. These findings corroborate Crites (1956), who suggested a range of 8–14 cuticular lamellae and should be considered in the identification key of the species according to Vieira *et al*. (2020).

Li (2019) analysed specimens of *C*. *americana* collected from the marsupial *D*. *virginiana* in the United States using light microscopy and scanning electron microscopy for the first time. The SEM images revealed the internal structures of the pharynx of *C*. *americana* and the presence of pharyngeal lamellae instead of pharyngeal teeth and/or teeth-like structures. In the present study, when analysing specimens of *C*. *tentaculata* by light microscopy and SEM, we observed that the structures in the pharynx are thin, flattened and parallel to each other. With this, we agree that these structures are actually cuticular lamellae, previously described as denticles or teeth-like structures.

Among the species of the genus *Cruzia*, only *C*. *tentaculata C*. *cameroni* and *C*. *americana* were reported parasitizing marsupials of the subfamily Didelphidae. *Cruzia americana* differs from *C*. *tentaculata* because it has 15–18 cuticular lamellae in each longitudinal line and 5 pairs of postcloacal papillae, while *C*. *tentaculada* has 4 pairs of postcloacal papillae. In addition, *C. tentaculata* has a single papilla on the anterior edge of the cloaca, but this papilla was not found in specimens of *C*. *americana* (Travassos, 1922; Ruiz, 1947; Wolfgang, 1951; Crites, 1956; Adnet *et al*., 2009; Li, 2019). Li (2019) also reported that the intestinal diverticulum of *C*. *americana* is distinctly longer than that of *C*. *tentaculata*. *Cruzia cameroni* can be differentiated from *C*. *tentaculata* because it has 13–17 cuticular lamellae and distinctly larger spicules and does not have a single papilla on the anterior edge of the cloaca. In addition, *C*. *cameroni* has the vulva located in the equatorial region of the body, while the vulva of *C*. *tentaculata* is located in the pre-equatorial region.

Regarding the discriminant analyses, significant differences in morphometric traits of *C. tentaculata* were observed in both males and females either among localities or among host species. We hypothesize that these differences are related to several factors related to the coevolutionary processes of host–parasite interactions. Differences in host body size or host metabolic rates would influence the size of some helminth traits because, for having an endoparasitic way of life, they must be adapted to the host characteristics. Differences among localities can be attributed to distinct local ecological factors, which in turn could influence the host characteristics, as well as the free-living stages of the helminth. Such a hypothesis could only be confirmed with a more comprehensive study encompassing all the host species in which this helminth has been found and including measurements of environmental variables and host attributes.

### Population structure of *Cruzia tentaculata* related to locality and hosts

The AMOVA showed a similar percentage of genetic variation between and within locations (51.58% and 48.42%, respectively). This fact is corroborated by the low and non-significant Fst values between localities, indicating low genetic differentiation, low geographic structure and high gene flow of the parasite populations among the studied areas. A mixture was found between the clusters (1, 3, 4 and 5), corroborating the haplotype network, the phylogenetic analyses and the low Fst values. In addition, cluster 2 recovered in BAPS was congruent with group II found in the haplotype network. However, the heterogeneity observed between populations can be attributed to geographic distance, corroborated by the Mantel test, which indicated a significant and high (68%) correlation between geographic and genetic distance, suggesting isolation by distance. Most of the genetic divergence, shown by haplotype networks, is between the northern populations and the southern ones, separated by 1200 km.

The phylogenetic analyses and the haplotype network showed that specimens of *C*. *tentaculata* were not correlated with the host species. In addition, the host species were also observed to share haplotypes. The pattern found in phylogenies was corroborated in the haplotype network, and no structure was observed associated with the host species of *C*. *tentaculata*, with AMOVA indicating greater genetic variation within each host species (88.85%). In addition, the Fst value was very low and significant and was more evident between *D*. *aurita* and *D*. *albiventris*. Didelphine marsupials are phylogenetically close and can occur in sympatry in regions of ecotone between the Atlantic Forest and the Cerrado, showing overlap in their diets, which allows parasite sharing and therefore sharing of their haplotypes (Leite *et al*., 1996). In population genetic structures, the host is a key element among parasite populations that determines genetic variability and gene flow (McCoy *et al*., 2003), and gene flow is dependent on host displacement and encounters; thus, parasites partially share the phylogeographic history of their host (Nieberding *et al*., 2004, 2005).

Furthermore, the generalist characteristic of *C. tentaculata* infecting a wide range of host species, such as *C. philander, D. marsupialis, Marmosa murinae, Metachirus myosuros, M. nudicaudatus, Monodelphis domestica* and *Philander opossum*, may contribute to the homogenization of populations. In addition, Nascimento *et al*. (2018) demonstrated that *D. albiventris* forms structured populations along the northeast/south-southeast axis (Nascimento *et al*., 2018), coinciding with the results found for *C. tentaculata*.

López-Caballero *et al*. (2015) conducted a study to analyse the population structure of the helminth *Oligacanthorhynchus microcephalus* (Rudolphi, 1819) (phylum Acanthocephala) in *Didelphis marsupialis*, *D*. *virginiana* and *Philander opossum* in central and southeastern Mexico. Phylogenetic analyses demonstrated a similar pattern to that of *C*. *tentaculata*, in which the specimens of *O*. *microcephalus* were not correlated with host species or geographical locality.

The high haplotypic diversity found in *C*. *tentaculata* was also identified in *Haemonchus contortus* (Rudolph, 1803), a parasite of sheep and goats (Dey *et al*., 2019). In the present study, of the 164 sequences of the MT-CO1 gene of *C*. *tentaculata,* 144 haplotypes with 161 polymorphic sites were corroborated by *H. contortus,* of which 85 sequences of the mitochondrial gene nad4 resulted in 77 haplotypes with 115 polymorphic sites. In addition, *H*. *contortus* showed a low Fst value, and high gene flow was observed among the subpopulations, similar to that observed in the analyses of *C. tentaculata*.

Our results showed that there is a possibility of having 2 lineages, 1 from the southeast (*D*. *aurita*) and another from the northeast (*D*. *albiventris*). The haplotype network showed the separation of the 2 groups. We believe that the centre of origin was in *D*. *aurita* (southeast) and that secondary infections infected *D*. *albiventris* (northeast), as shown in the haplotype network.

### Demographic and biogeographic history of *Cruzia tentaculata*

Historical events leave signatures in the DNA, and neutrality tests can infer the demographic history of populations. Significantly negative values in neutrality tests indicate that the population is in an expansion process, showing a large number of rare haplotypes. Significant positive values in neutrality tests indicate that the species has experienced a population bottleneck, that is, the lack of rare haplotypes due to a drastic reduction in population size (Hartl and Clark, 2010). In the neutrality tests of the different groups studied, some of them presented significant negative values. Considering Fu's *F*s related to the hosts *D. aurita* and *D. albiventris* and for most of the locations, the results indicated the occurrence of an expansion process, although not corroborated by Tajima's *D*. However, the signature of population expansion was corroborated by the genetic diversity indices that were performed for the localities and host groups. High haplotypic diversity and low nucleotide diversity were found for the MT-CO1 gene; that is, although there were a large number of haplotypes, they differed from each other by a few nucleotide substitutions. This finding indicates a recent population expansion (Avise, 2000).

Pôrto *et al*. (2004) carried out an integrated study encompassing water resources, socioeconomic aspects, fauna, flora and environmental education, particularly regarding the biodiversity of highland humid forests in the northeast Atlantic Forest (Paraíba e Pernambuco states), and reported the occurrence of endemic species as well as Amazonian species. The coastal forest in the Brazilian northeast has been identified as an important centre of endemism in South America, which is influenced by the Amazonian biota in the north (Prance, 1982, 1987) and the Atlantic Forest in southern and southeastern Brazil (Andrade-Lima, 1960, 1982). The authors reported a hypothesis for the origin of the phytophysiognomy of highland humid forests associated with climatic variations that occurred during the Pleistocene, which allowed the Atlantic Forest to occupy the Caatinga domains, followed by interglacial periods, and the formation of Atlantic Forest islands in the Caatinga domains named ‘refuges of diversity’ (Andrade-Lima, 1982). In fact, our results corroborated this hypothesis since we observed a population isolation of *C. tentaculata* coinciding with the historical aspects suggested for the constitution of the northeastern Atlantic Forest.

The ‘Hypothesis of refuges’ predicts that during the glacial periods, the flora and fauna of forested areas became isolated in different forest fragments (refuges), differentiating and leading to speciation. When the glacial period ended, the forests expanded, and the species met again. The Atlantic Forest has 5 areas of great genetic diversity, which correspond to forest refuges (Carnaval and Moritz, 2008). They are (1) a great refuge from the north of Espírito Santo state to the northern coast of Bahia state; (2) a large refuge in the southeast region, including the south of Rio de Janeiro state, the northeast of São Paulo state and the southeast of Minas Gerais state; (3) a small area on the coast of Alagoas and Pernambuco states; (4) a small area in Chapada Diamantina, in the interior of Bahia state; and (5) a small area in northeastern Mato Grosso do Sul state. According to Carnaval and Moritz (2008), in non-refugee areas, molecular signatures of recent expansion are expected to be found. In our analyses, the groups recovered in the haplotype networks were distributed in regions that did not correspond to refuges, which suggests the expansion of 2 lines of *C*. *tentaculata* isolated from the forest areas of refuges that expanded to other regions during interglacial periods. These findings suggest that 1 of these lineages of *C. tentaculata* may have migrated from the northeast (Sergipe and Paraíba states), giving rise to group I. The second lineage may have differentiated and expanded by the northeast region (Sergipe state), southeast (Rio de Janeiro and Minas Gerais states) and dispersed to the south (Paraná and Rio Grande do Sul states), giving rise to group II.

## Conclusions/future directions

Some analyses, such as the Mantel test, AMOVA, the low values of Fst and the sharing of haplotypes, indicate that there is a high gene flow between the subpopulations of the Atlantic Forest, corroborating our initial hypothesis that the specimens of *C*. *tentaculata* may represent a panmictic population along the Atlantic Forest. However, we cannot affirm that the analysed individuals of *C*. *tentaculata* form a panmictic population, which is supported by the neutrality tests, morphometric differences found, and the haplotype networks, which showed the existence of 2 groups. Thus, despite the low genetic differentiation observed, the first hypothesis cannot be confirmed with the data of the present study, unlike the second hypothesis, which was corroborated. We suggest that the morphological differences and the haplotypes grouping into 2 groups observed are attributed to ecological differences, which can be explained by the hypothesis of refuges. We suggest a more comprehensive study including other molecular markers to corroborate the observed patterns and the inclusion of parasite samples from the Cerrado and Amazon biomes to test the hypothesis of refuges.

## Supplementary material

For supplementary material accompanying this paper visit https://doi.org/10.1017/S0031182022000981. click here to view supplementary material
