# Supplementary figures and images for: Population genetic structure and morphological diversity of Cruzia tentaculata (Nematoda: Ascaridida), a parasite of marsupials (Didelphinae), along the Atlantic Forest on the eastern coast of South America
Source: Parasitology. 2022 Jul 13;149(11):1487–504. doi: 10.1017/S0031182022000981 (PMC10090786; doi:10.1017/S0031182022000981)

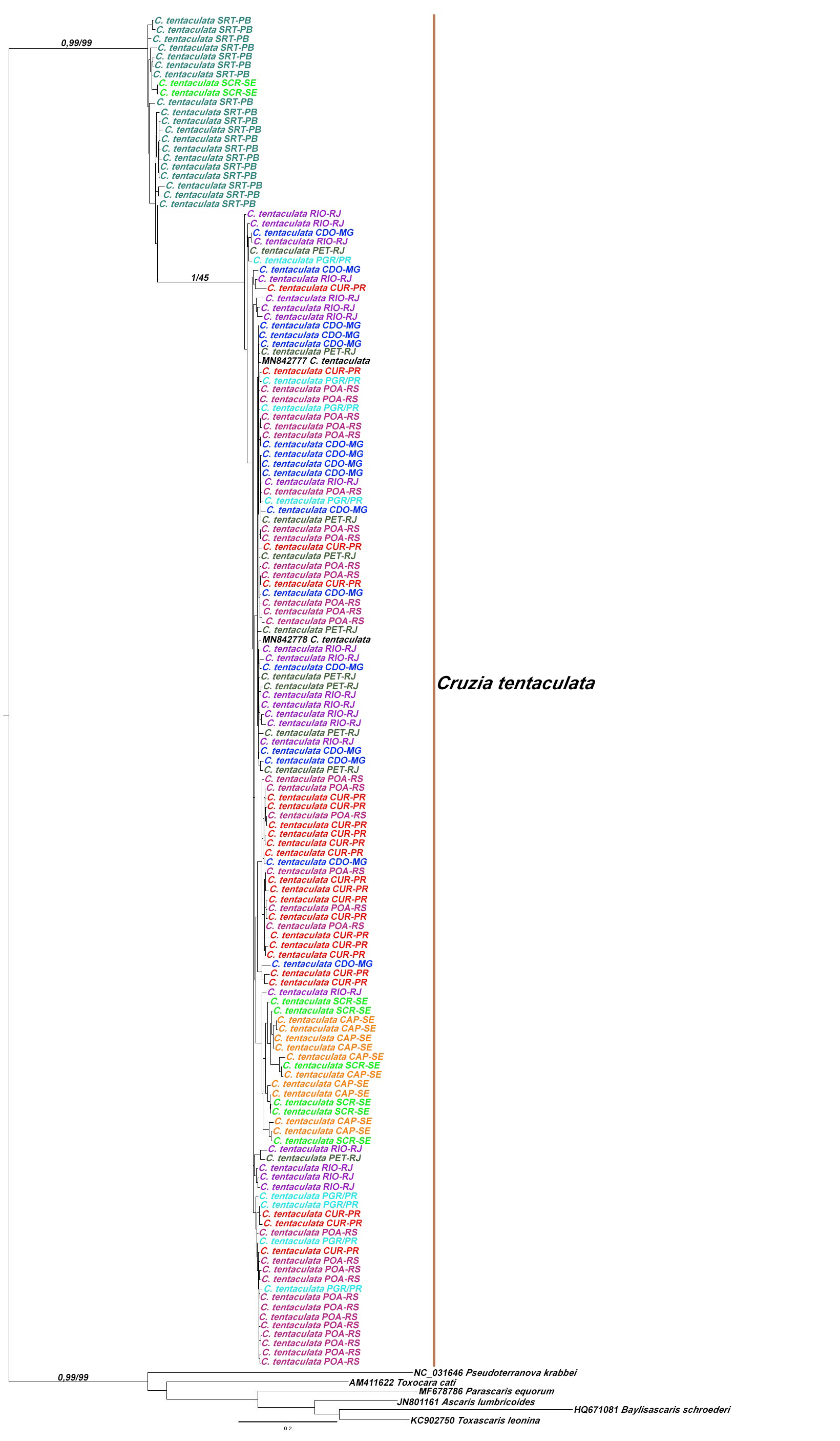

Supplement: Supplementary file 1 [file S0031182022000981sup001.tif]
